# Epidemiological surveillance of bovine viral diarrhea and rift valley fever infections in camel

**DOI:** 10.14202/vetworld.2018.1331-1337

**Published:** 2018-09-26

**Authors:** Halla E. K. El Bahgy, Hala K. Abdelmegeed, Marawan A. Marawan

**Affiliations:** 1 Department of Hygiene and Veterinary Care, Faculty of Veterinary Medicine, Benha University, Qualyobia, Egypt; 2 Department of Virology, Animal Health Research Institute, Doki, Giza, Egypt; 3 Department of Animal Medicine (Infectious Diseases) Faculty of Veterinary Medicine, Benha University, Qualyobia, Egypt

**Keywords:** bovine viral diarrhea virus, epidemiological, rift valley fever virus, risk factors, sequencing, surveillance

## Abstract

**Aim:**

This study was designed to investigate the current epidemiological situation of bovine viral diarrhea virus (BVDV) and rift valley fever virus (RVFV) infection of camels originating from Sudan “smuggler” and Egypt as part of our future plan for a national surveillance program in Egyptian provinces, which will aid in establishment of control strategy for animal diseases.

**Materials and Methods:**

This investigation was accomplished using serological diagnostic and molecular biology techniques. A total number of 200 blood samples were collected from camel (120 originated from Sudan “smuggler” and 80 from local breed) and were subjected for testing both BVDV and RVFV occurrence with different age and sex.

**Results:**

Sixty-six of the 200 camels (33%) were positive for BVDV antibodies, and 44 (22%) for BVDV antigen (Ag), and 27 of the 200 camels (13.5%) were positive for RVFV immunoglobulin G (IgG) antibodies. On the other hand, the seroprevalence of BVDV for antibodies (47.5%), Ag (31.6%), and RVFV IgG antibodies (16.6%) was higher in camel originated from Sudan “smuggler” than of local breed which was 11.2% for BVDV antibodies and 7.5% for BVDV Ag, while it was 8.7% for RVFV IgG antibodies. The incidence of BVDV antibodies, Ag, and RVFV IgG antibodies was the highest in male, up to 9 years of age. The frequency of positive cases was significantly different according to the origin of samples and sex and age of camel for BVDV and RVFV. In addition, seven serologically positive samples for BVDV and five serologically positive samples for RVFV were submitted as a buffy coat for molecular detection by one-step – reverse transcription polymerase chain reaction (RT-PCR). The results demonstrated that three samples were positive for BVDV of camel originated from Sudan (smuggler), while no RVFV Ag was detected in all five samples. Sequencing and phylogenetic analysis of the amplicons obtained from positive RT-PCR samples (three samples) indicated 100% nucleotide homology with Sudan strain 2015 except only one (missense point mutation) by substitution of A to T at position 345 that changed the coded amino acids from T (Threonine) to S (Serine) at residue 115.

**Conclusion:**

Camels act as risk animals for the introduction of many infectious diseases from Sudan to Egypt, especially transboundary animal diseases, so strict quarantine measures should be taken during importation of live animals from Sudan to prevent the spread of such diseases.

## Introduction

Camels are considered an important source for human food, transportation, and entertainment, especially in the Arabian countries [1]. Camels are susceptible to many viral diseases. It plays an important role in epizootiology of many diseases and amplification of some viruses [2]. Contrary to the OIE rules, camels enter Egypt officially without any virological investigation or sufficient period for quarantine. The continuous importation of viremic camels, from Sudan, acts as the main source of many critical viral outbreaks in Egypt [1,3].

Bovine viral diarrhea virus (BVDV) and rift valley fever virus (RVFV) have the similar potential outcome in causing reproductive and respiratory manifestation in camel [4,5]. BVDV is a member of the family *Flaviviridae*, genus *Pestivirus*, single-stranded linear RNA [6] that crosses the placenta in pregnant cows which are infected between days 40 and 125 of the gestation period causing a variety of disease syndromes, including reproductive losses due to abortion, stillborn calves, or calves that die early in life and birth of persistently infected. Moreover, RVFV is a member of the family *Bunyaviridae*, genus *Phlebovirus* (80-110 nm in diameter), 3-segmented (L, M, and S), single-stranded negative-sense RNA genome, and single serotype [7] that is an acute arboviral (insect-borne) disease, mainly affecting ruminants and human, causing abortion in pregnant animals at the late stage of pregnancy and high mortalities among young animals [8].

Bovine viral diarrhea is one of the important viral diseases causing respiratory distress and abortion, while rift valley fever is one of the most serious zoonotic transboundary animal diseases. The World Organization for Animal Health (OIE) defined them as a communicable disease which has the potential for serious and rapid spread, irrespective of national borders. They have a serious economic impact on the animal production sector as causing losses from reproductive failures and increase the severity of secondary infections by other pathogens [9].

In Egypt, BVD was considered as an endemic disease before 1992. BVD virus was first isolated in 1975 from calves suffering from severe enteritis [10], and since then, the disease continues to be recorded not only in cattle and buffaloes but also in other species of animals including sheep, goats, and camels in several provinces of Egypt [11]. BVDV was previously isolated as the Egyptian strains (Iman and Kenna) in 1975 and 1982, respectively, and was typed as CP-BVDV 1 and the strain of Behera-CP 58/99 that isolated from the milk and identified as CP-BVDV 2 [11,12]. The first report of a BVDV-2 infection in dromedary camels in Egypt was reported by Yousif et al. [13]. On the other hand, RVF virus was firstly identified during an investigation of an epizootic occurred among sheep on a farm in the Rift Valley of Kenya in 1931 [14]. RVF virus invaded Egypt for the first time in 1977 in Zagazig province causing abortion in about 20% of all pregnant domestic animals and deaths in young lambs [15]. Later on, the next documented RVF infection was recorded in early 1981 [16], and 12 years later, RVF was noted in man and domestic animals in Egypt in Aswan Governorate in late May 1993 [17]. RVF outbreaks occurred again in Egypt between April and August 1997 in Aswan and Assiut Governorates, and the signs appeared among infected cattle and sheep were high fever, icterus, bloody diarrhea, and abortion [3].

The majority of herd diagnosis for the detection of BVDV and RVFV infections is often based indirectly on an interpretation of serological tests of animal screenings. Although there are some reports on BVDV and RVFV infection and genotyping of the isolated viruses in Egypt, still there are no appropriate data on surveillance and percentage of infection because those reports are often based on selected cases. This makes it difficult to estimate the real percentage of the disease in animals [10].

There are various methods available for diagnosing acute or persistent BVDV infections. These include immunohistochemistry, several antigens (Ags) and antibody enzyme-linked immunosorbent assays (ELISAs), reverse transcription polymerase chain reaction (RT-PCR) assays, and virus isolation [18]. The serological diagnosis of RVF virus infection was based on the plaque reduction neutralization test and hemagglutination inhibition test. The complement fixation test and indirect immunofluorescence assay have also been used for detecting RVF viral antibodies. Recently, the ELISA has been adapted for this purpose [19]. A rapid diagnosis can also be achieved by molecular detection of viral RNA using validated conventional or RT-PCR. These techniques should be followed by sequencing of the selected sample [20]. In the present study, BVDV and RVFV were diagnosed serologically using ELISA for detection of their specific antibodies as well as both viruses antigen were detected molecularly using RT-PCR followed by sequential analysis.

The present study was designed to investigate the current epidemiological surveillance of BVDV and RVFV infections of camels originating from Sudan “smuggler” and local breed as part of our future plan for a national surveillance program throughout the country, which will aid in controlling both diseases. This investigation was accomplished using serological and molecular diagnostic techniques.

## Materials and Methods

### Ethical approval

This study was approved by the Research Committee of the Faculty of Veterinary Medicine, Benha University, Qualyobia, Egypt.

### Sampling

A total number of 200 blood samples were collected from camel (120 originated from Sudan “smuggler” and 80 originated from different herds in Egypt “local breed”) and were subjected for the investigation of BVDV and RVFV infections with different age and sex. In addition, 20 buffy coat samples (ten samples from smuggler camel and ten from local breed) were collected from febrile animals and stored at −20°C for molecular detection of BVDV and RVFV Ags under the national and international standard biosafety conditions and ethics.

The clear serum was obtained using sterile Pasteur pipettes from the blood samples were left in tightly closed tubes overnight at 4°C and then centrifuged at 3000 rpm for 10 min to separate the sera and non-sera fractions. The clear serum placed in Eppendorf tubes, labeled, and stored at −70°C in the laboratory for BVDV Ag, BVDV antibodies, and IgG antibodies of RVFV using ELISA test as serological test.

### Serological tests

#### BVDV antibody detection by competitive ELISA

Antibodies detection against BVDV was performed on camel serum samples using the commercial kit, (INgezim BVD^®^ compact ELISA, Ingenasa, Spain) according to the manufactures’ instruction.

#### BVDV Ag detection by Ag-capture ELISA (ACE)

Ag detection of BVDV was performed on camel serum samples using the commercial bovine viral diarrhea virus Ag kit/serum plus IDEXX HerdChek BVD of Ag/serum plus ELISA test according to the manufacturer’s instruction.

#### RVFV-specific IgG detection by competitive ELISA

Anti-RVFV nucleoprotein antibodies were detected by ID screen^®^ RVF competition multispecies ELISA kit (ID.Vet, France). RIFTC VER 0411 GB, ID according to the manufacturer’s instruction.

### Molecular detection of BVDV NS2-3 protein using one-step RT-PCR

The viral RNA was extracted from buffy coat samples (ELISA-positive samples) by QIAamp^®^ Viral RNA Mini Kit (Qiagen, Hilden, Germany) following the mini spin protocol according to the manufacturers’ instructions. About 1 μL of the obtained RNA was used as the template in a one-step RT-PCR (Ready-To-Go RT-PCR Beads, Amersham).

The primers used were forward primer O1100 5’-CATGCCCTTAGTAGGACTAGC-3’ and reverse primer Ol 380R 5’-AACTCCATGTGCCATGTACAG-3’[21].

The amplification reaction was carried out using Thermal Cycler (Life ECO, China) in the following cycles: The first step was the reverse transcription step, this step was carried out at 50°C for 30 min for one cycle, then followed by initial denaturation at 94°C for 15 min for one cycle. After that 40 cycles of amplification in three steps consisting of denaturation at 94°C for 30 s, annealing at 55°C for 30 s, and extension at 72°C for 30 s were performed, finally one cycle of final extension at 72°C for 10 min then holding at 4°C∞. The PCR products were placed on a gel plate prepared using Tris–EDTA buffer containing 1.5% agarose gel, stained by 2 μLGelRed™ stain (10.000× in DMSO Cat # 41002, Biotium, Hayward, USA) then electrophoresed at 100 V for 30 min, and finally visualized by ultraviolet light transilluminator (BioRAD) after using a 50 bp DNA ladder.

### PCR product extraction from the gel and purification

The obtained RT-PCR products were purified by Qiaquick PCR Purification Kit (Qiagen) according to the manufacturers’ instructions. The RT-PCR products were eluted in nuclease-free water to be ready for sequencing purpose.

### Sequential analysis

Sequence analysis was carried out to detect the nucleotide and amino acid composition of the detected strain. It was carried out in both directions using the previously mentioned forward primer and reverse primers by 3730 DNA Analyzer, Applied Biosystems, USA. BigDye Terminator v3.1 Cycle Sequencing Kit (Applied Biosystem, UK) was used as recommended by the manufacture protocol with small modifications

#### Multiple sequence alignment and phylogenetic analysis

Multiple sequence alignment and phylogenetic analysis were applied for sequence alignment and construction of phylogenetic tree to detect the genetic relatedness of the tested strains compared to other worldwide strains registered in the GeneBank.

The nucleotide sequence data obtained after genetic analysis in DNA Analyzer “286 nucleotide sequences were used in analysis,” and the data from others *BVDV NS2-3* protein sequences from different localities registered in GeneBank were compared, aligned, and analyzed using BioEdit Software Program V.5.0.9, USA [22].

Phylogenetic analysis was carried out by constructing neighbor-joining (NJ) tree of the nucleotide sequence data using the MEGA 7 software program with the evolutionary model set to Kimura 2-parameter + Gamma. The reliability of the phylogenetic relationships was evaluated using non-parametric bootstrap analysis with 1000 replicates for NJ analysis [23,24].

### Molecular detection of RVFV s gene using one-step RT-PCR

The viral RNA was extracted from buffy coat samples (ELISA-positive samples) by QIAamp^®^Viral RNA Mini Kit (Qiagen, Hilden, Germany) following the mini spin protocol according to the manufacturers’ instructions. About 1 μL of the obtained RNA was used as the template in a one-step RT-PCR (Ready-To-Go RT-PCR Beads, Amersham).

The primers used were forward primer 5’-CCTTAACCTCTAATCAAC-3’ and reverse primer 5’-TATCATGGATTACTTTCC -3’ [25].

The amplification reaction was carried out using Thermal Cycler (Life ECO, China) in the following cycles: Reverse transcription was applied at 50°C for 30 min and a primary denaturation step was done at 95°C for 5 min, followed by 35 cycles of 94°C for 30 s, 55°C for 40 s, and 72°C for 50 s min. A final extension step was done at 72°C for 10 min. The PCR products were placed on a gel plate prepared using Tris–EDTA buffer containing 1.5% agarose gel, stained by 2 μLGelRed™ stain (10.000X in DMSO Cat # 41002, Biotium, Hayward, USA), then electrophoresed at 100 V for 30 min, and finally visualized by ultraviolet light transilluminator (BioRAD) after using a 50b pDNA ladder.

### Statistical analysis

The statistical analyses were performed by univariate analysis of variance, one-way ANOVA, and independent t-tests using SPSS program version 20 (IBM, USA). p<0.05 was assumed for statistical significance.

## Results

The results of the serological survey for BVDV and RVFV from camels are shown in Table-1. Sixty-six of the 200 camels (33%) were positive for BVDV antibodies and 44 (22%) for BVDV Ag, and 27 of the 200 camels (13.5%) were positive for RVFV IgG antibodies. The seroprevalence of BVDV for antibodies (47.5%), Ag (31.6%), and RVFV IgG antibodies (16.6%) was higher in camel originated from Sudan “smuggler” than camel local breed which was 11.2% for BVDV antibodies and 7.5% for BVDV Ag, while it was 8.7% for RVFV IgG antibodies. The incidence of BVDV was the highest (48.5%) for antibodies and 34.2% for Ag in male camel, in addition to 60% for antibodies and 40% for Ag in older age up to 9 years. On the other hand, the incidence of RVFV was the highest (21.4%) in male camel and 25.7% in older age up to 9 years.

**Table-1:** Incidence of seropositive camels for BVDV antibody, antigen, and RVFV antibodies (IgG) according to some risk factors.

Risk factors	Number of samples	BVDV antibody	BVDV antigen	RVFV antibody (IgG)
		
		Number of positive (%)	Number of positive (%)	Number of positive (%)
Origin				
Sudan	120	57 (47.5)	38 (31.6)	20 (16.6)
Local breed	80	9 (11.2)	6 (7.5)	7 (8.7)
Sex				
Male	70	34 (48.5)	24 (34.2)	15 (21.4)
Female	130	32 (24.6)	20 (15.3)	12 (9.2)
Age (years)				
≤1	26	11 (42.3)	7 (26.9)	5 (19.2)
2-5	60	3 (5)	1 (1.7)	2 (3.3)
5-9	79	31 (39.2)	22 (27.8)	11 (13.9)
≥9	35	21 (60)	14 (40)	9 (25.7)
Total	200	66 (33)	44 (22)	27 (13.5)

BVDV=Bovine viral diarrhea virus, RVFV=Rift valley fever virus, IgG=Immunoglobulin G

The frequency of positive cases was significantly different between origin of samples and sex and age of camel for BVDV and RVFV as shown in Table-2.

**Table-2:** Frequency of BVDV and RVFV seropositive in camel herds (n=200) in relevance to different investigated factors.

Risk factors	BVDV antibodies	BVDV antigen	RVFV antibody (IgG)
Origin			
Sudan	3.47±0.04^a^	1.23±0.04^a^	1.16±0.03^a^
Local breed	3.11±0.03^b^	1.07±0.02^b^	1.08±0.03^b^
Sex			
Male	1.48±0.06^a^	1.34±0.05^a^	1.21±0.04^a^
Female	1.24±0.03^b^	1.15±0.03^b^	1.09±0.02^b^
Age (years)			
≤1	1.42±0.09^ab^	1.26±0.08^a^	1.19±0.07^a^
2-5	1.05±0.02^c^	1.01±0.01^b^	1.03±0.02^b^
5-9	1.39±0.05^b^	1.27±0.05^a^	1.13±0.03^ab^
>9	1.60±0.08^a^	1.42±0.08^a^	1.25±0.07^a^

The means with different superscripts in the same column indicate significant difference; the significant difference is at the <0.05 level. BVDV=Bovine viral diarrhea virus, RVFV=Rift valley fever virus, IgG=Immunoglobulin G

From the febrile camel, seven buffy coat samples of BVDV-positive serological analysis and five buffy coat samples of RVFV-positive serological analysis were submitted for rapid detection by one-step RT-PCR. We found that three samples of seven were positive with RT-PCR for BVDV of camel originated from Sudan (smuggler) and successfully amplified 286 bp corresponding to the BVD genome. On the other hand, no RVFV Ag was detected in all five samples.

The amplified amplicons of BVDV RT-PCR-positive samples were extracted from the gel after electrophoresis, then purified, and finally succumbed to sequential analysis. The sequence analysis was carried out using BigDye termination method in both directions using the above-mentioned forward and reverse primer, and then, the result was analyzed using 3730 DNA analyzer.

After direct sequencing, 286-nucleotide sequences corresponding to partial *BVDV NS2-3* encoding gene (from nucleotides 105 to 390) and amino acid sequences (from 35 amino acids to 130) of the full-length BVD genome were aligned and analyzed.

The obtained data revealed 100% nucleotide homology with Sudan strain 2015 except only one (missense point mutation) by substitution of A to T at position 345 that changed the coded amino acids from T (Threonine) to S (Serine) at residue 115.

**Figure-1:**
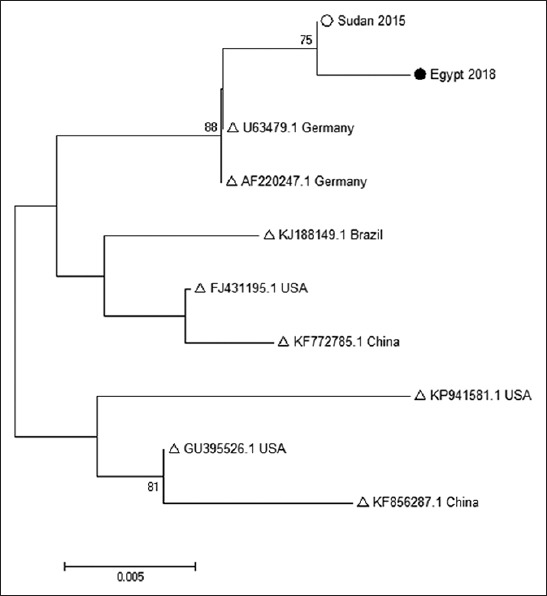
Phylogenetic tree of the bovine viral diarrhea (BVD) strain. The phylogenetic tree was based on partial sequences (286 nucleotides) of the BVD virus NS2-3 encoding gene. The sequence from this study labeled with black circle while Sudan strain with white circle and from Genbank labeled with white triangle. The evolutionary history was inferred using the neighbor-joining method with bootstrap probability more than 70%. The bootstrap values, more than 70%, are shown next to branches. The tree is drawn to scale, with branch lengths in the same units as those of the evolutionary distances used to infer the phylogenetic tree.

Phylogenetic analysis was carried out to detect the genetic relatedness of the isolated strain in the current study with other strains worldwide expressed as accession numbers (Figure-1).

Phylogenetic analysis was carried out by constructing NJ tree of the nucleotide sequence data using the MEGA 7 software program with the evolutionary model set to Kimura 2-parameter + Gamma. The reliability of the phylogenetic relationships was evaluated using non-parametric bootstrap analysis with 1000 replicates for NJ analysis.

The results of the phylogenetic analysis indicated that the isolated strain in this study was clustered with Sudan strain, proving the same source of infection.

## Discussion

Surveillance is an important defense in our country’s resilience against animal diseases. It is the continuous monitoring of the occurrence of disease within a population through the collection, collation, analysis, and dissemination of disease-related data. This information is used as a guide to the management of diseases within populations through planning and evaluating strategy measurement to prevent and control disease. Animal surveillance has and will continue to be an important component of the veterinary infrastructure supporting livestock industries, food production systems, and rural economies around the world.

Camels may play a role in the persistence and transmission of many diseases through direct contact with domestic animals, such as sheep and goats, and to a lesser extent with cattle in Egypt, especially BVDV and RVFV diseases [26,27].

In a serological survey, the incidence of BVDV antibodies and Ag in 200 camels tested was 47.5% for antibodies, and 31.6% for Ag in camel originated from Sudan, but in local breed camels, it was 11.2% for BVDV antibodies and 7.5% for BVDV Ag. These findings indicated that there is a failure in the quarantine measures which are applying for live camels exporting from Sudan. The previous findings disagree with the findings of Saidi *et al*. [28] who found that the seropositivity rate was 9.0% for BVDV-specific antibody, although 41.4% of camels tested were positive for BVDV Ag. Moreover, the results agreed with a study of Wernery [29] who recorded that neutralizing antibodies was 11% to BVDV in Egypt, with a peak 23% in one area. In another Egyptian survey, Tantawi *et al*. [30] detected 4.3% BVDV-positive dromedaries and disagreed with the results of Zaghawa [31] who found that camels from Egypt exhibited an even higher prevalence (52.5%) of neutralizing antibodies to BVDV.

On the other hand, the incidence of RVFV IgG antibodies was 16.7%, and 8.7% in the examined camels originated from Sudan and local breed, respectively. There are previous studies in Egyptian camels during the present interepidemic period which was conducted in 2009/2010 with 10 camel sera from an abattoir and in 2012 encompassing 100 camels from Qualyobia Governorate. Likewise, no further information about the origin of the animals was given in these reports. Both studies failed to detect antibodies in Egyptian camels. Only 10% of imported animals are tested against RVFV antibodies directly after the import as a routine disease control [32]. The risk to import viremic animals should not be neglected; hence, continuing investigations on the role of camels should be in focus of further investigations. These findings agreed with the results of El-Harrak *et al*. [33] who found that 15 of 100 camel serum samples were positive for RVFV-specific IgG antibodies by competitive ELISA.

The incidence of BVDV antibodies, BVDV Ag, and RVFV IgG antibodies was the highest in the age group of ≥9 years. Furthermore, there is a highly significant correlation between the effect of age and incidence of BVDV antibodies, BVDV Ag, and RVFV IgG antibodies in the examined camel serum samples. This might indicate that older animals have a higher exposure to infection and contact could have occurred some years ago [33,34].

The proportion of the positive camels for BVDV antibodies, Ag, and RVFV IgG antibodies were the highest in male than female. There was a statistical difference between the sexes with BVDV antibodies, Ag, and RVFV IgG antibodies in the examined camel serum samples. This may be due to the fact that male camels travel from one place to another place to provide transportation service more than female camels so that they have a higher probability of acquiring an infection. Frequent travel could also compromise their immune response to infection due to the stress of fatigue. These findings disagreed with the results of Raoofi *et al*. [34] who found that the frequency-positive cases were not significantly different between male and female.

The results indicated that camels might represent an important source for BVDV infection in all ruminants, including cattle, sheep, and goats breed in mixed herds since they had higher BVDV prevalence rates. Therefore, the prevention and control measures for BVDV spreading and persistence the camel should house in camel populations only to limit the spread of BVDV infection to ruminant populations [28]. The results of phylogenetic analysis of BVDV indicated that the isolated strain in this study was clustered with Sudan strain, proving the same source of infection.

Camels consider as the contagious population in Egypt so play an important role in the transmission of many diseases, especially BVDV and RVFV, throughout traveling of camel from country to another country. Based on these study findings, continuous disease surveillance of camels for BVDV and RVFV is indicated, which is a good tool for eradication, control of such infectious diseases, and measuring RVFV circulating activity in non-vaccinated camels. Finally, the difference in the prevalence among countries may due to numbers of animal used, occurrence of vector, and previous factors such as vaccination and quarantine measures.

## Conclusion

Camels act as risk animals for the introduction of many infectious diseases from Sudan to Egypt, especially transboundary animal diseases, so strict quarantine measures should be taken during importation of live animals from Sudan to prevent the spread of such diseases. The control of such diseases was submitted to main points as routine annual surveillance, vaccination of animals, and vector control.

## Authors’ Contributions

HEKE, HKA, and MAM designed the concept for this research and scientific paper. HEKE has conducted the maintenance of camels used in the experiment, collecting samples, and compiling the resource materials. HKA provided technical supports and made serological examination of serum samples. HEKE analyzed the serological data. MAM conducted the molecular detection of BVDV and RVFV in buffy coat sample and sequencing of BVDV. All authors participated in the manuscript’s draft and revision. All authors have read and approved the final manuscript.
